# One-step Synthesis of Few-layer WS_2_ by Pulsed Laser Deposition

**DOI:** 10.1038/srep18116

**Published:** 2015-12-11

**Authors:** Tamie A. J. Loh, Daniel H. C. Chua, Andrew T. S. Wee

**Affiliations:** 1 Department of Materials Science and Engineering, National University of Singapore, 7 Engineering Drive 1, Singapore 117574, Singapore; 2 Department of Physics, National University of Singapore, 2 Science Drive 3, Singapore 117542.

## Abstract

Atomically thin tungsten disulfide (WS_2_) has attracted much attention in recent years due its indirect-to-direct band gap transition, band gap tunability, and giant spin splitting. However, the fabrication of atomically thin WS_2_ remains largely underdeveloped in comparison to its structural analogue MoS_2_. Here we report the direct fabrication of highly crystalline few-layer WS_2_ on silver substrates by pulse laser deposition at the relatively low temperature of 450 °C. The growth takes places by conventional epitaxy, through the *in-situ* formation of nearly lattice-matching Ag_2_S on the silver surface. Intriguingly, it was observed that the resulting film was composed of not only the usual semiconducting 2H-WS_2_ structure but also the less common metallic 1T-WS_2_. Modifications of the synthesis parameters allow for control over the crystalline quality, film thickness and crystal phase composition of the resulting WS_2_ film.

Among the many materials that have the capacity to form 2-dimensional (2D) layers, much attention has been devoted to the lamellar transition metal dichalcogenides (TMDC)^1^,^2^,^3^,^4^. This class of materials, in which a plane of metal atoms are sandwiched between two planes of chalcogen atoms, exhibits interesting and diverse properties, ranging from insulators to semiconductors to metals^2^. As a typical example of a TMDC, tungsten disulphide (WS_2_) demonstrates a unique combination of structural, electronic, optical, mechanical, chemical, and thermal properties that have been the focus of various studies. Its electronic band gap for example, undergoes an indirect (1.4 eV) to direct (2 eV) transition when its size is reduced from bulk to a single layer^2^. The material has been used in fluorescent emitters^5^ and field effect transistors^6^, for photovoltaics^7^ and photocatalysis^8^, and is regarded as highly valuable for high power applications such as solid state batteries^9^ and supercapacitors^10^. Monolayer WS_2_ also possesses strong spin–orbit induced electronic band splitting and spin–valley coupling^11^,^12^, in addition to a band structure that can be tuned by variation in strain^13^. Until recently, most of the attention has been focused on the semiconducting phase of WS_2_ and MoS_2_, both of which possess a prismatic coordination for the metal atom (2H). This 2H-structure is generally produced by physical exfoliation or vapour growth methods. In contrast, the metallic 1T-phase, with an octahedral coordination for the metal atom, has been synthesised primarily through lithium intercalation. Both 1T-WS_2_ and 1T-MoS_2_ layers have recently been shown to be very efficient hydrogen evolution electrocatalysts^14^,^15^,^16^.

Unlike related systems such as MoS_2_, techniques for effective preparation of 2D WS_2_ remains largely underdeveloped. There are currently two main methods of preparing ultrathin WS_2_: (1) top-down exfoliation and (2) bottom-up substrate growth. Exfoliation can be mechanical^17^,^18^, chemically assisted^19^ (e.g. sonication in a good solvent) or purely chemical^14^,^20^ (intercalation with e.g. lithium), but the end results remain similar: high quality flakes can be prepared but there is very little control over their size, shape or nature of their edges. On the other hand, the substrate growth technique, commonly some form chemical vapour deposition^21^,^22^,^23^ (CVD), is capable of producing large area monolayers with high crystallinity and good control over flake shape. However, many of these substrate growth techniques utilize different solid precursors heated to high temperatures in the range of 750–1000 °C and require long growth times. Although pulsed laser deposition (PLD) is also a type of bottom-up substrate growth technique, it is unique in that it is a purely physical method. Among its advantages is the ability to grow high quality films, to ablate any material and to obtain a stoichiometric transfer of target material onto the substrate, which is especially useful in the case of composite materials such as WS_2._ While PLD has found success in the fabrication of 2D materials such as few-layer graphene^24^ and MoS_2_^25^, it remains underdeveloped compared to exfoliation and CVD methods.

In this work, we describe the synthesis of highly crystalline few-layer WS_2_ on Ag substrates using PLD at a relatively low temperature of 450 °C. Ag metal was selected as substrate for the ease of the ease of producing metal-semiconductor contacts without film transfer. Additionally, the introduction of a metal support can substantially alter the H binding energy of 2D TMDCs^26^, enabled by charge transfer from the substrate to the overlying film and through strong interactions at the interface. Metal supported WS_2_ could thus potentially be used as novel catalysts for hydrogen production. Surprisingly, using PLD to grow WS_2_ does not produce the expected semiconducting prismatic 2H structure. Instead, it creates metallic WS_2_ films with the distorted octahedral 1T-WS_2_ structure. The structure and properties of these films were explored through a combination of Raman spectroscopy, photoluminescence (PL) measurement, x-ray photoelectron spectroscopy (XPS), high-resolution transmission electron microscopy (HRTEM), and powder x-ray diffraction (PXRD).

## Results and Discussion

**Figure 1:**
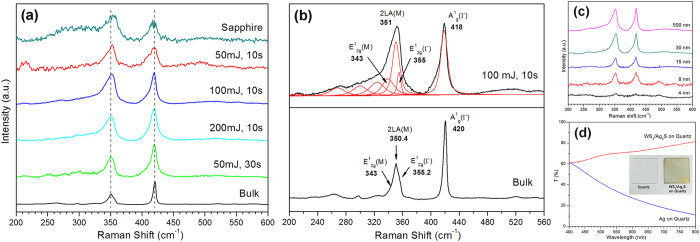
(**a**) Raman spectra of as-grown samples fabricated on Ag and sapphire. The WS_2_ film on sapphire was fabricated at 200 mJ and 10 s. The left and right dashed lines indicate the positions of the 2LA (M) and A_1g_ phonon modes in bulk WS_2_ respectively. (**b**) Multi-peak Lorentzian fitting of Raman bands in the Ag sample fabricated at 100 mJ and 10 s, with bulk WS_2_ included for comparison. (**c**) Raman spectrum of as-deposited WS_2_ on quartz with varying Ag buffer thickness. All samples were synthesized using the parameters of 100 mJ and 10 s. (**d**) Optical transmittance of a quartz substrate with Ag buffer layer of 8 nm, before and after WS_2_ film growth. Inset: Photograph of the as-grown WS_2_ sample and bare quartz.

Figure 1a shows the Raman spectra of the as-grown WS_2_ film on Ag excited under ambient conditions, with the spectrum of bulk WS_2_ and a WS_2_ sample grown on insulating sapphire substrates included for comparison. The spectrum of the sapphire sample was normalized to remove the peaks from the substrate. At this excitation wavelength of 514.5 nm, the spectrum reveals many second-order Raman peaks, in addition to the first-order phonon modes. The strongest peak at ~352 cm^−1^ can be resolved by multi-peak Lorentzian fitting into three individual contributions at 343, 351, and 355 cm^−1^ as shown in Fig. 1b for the sample fabricated at 100 mJ and 10 seconds. These modes are assigned to the in-plane vibrational E^!^_2g_ (M) mode, the second-order mode of longitudinal acoustic phonon 2LA (M), and the in-plane vibrational E^!^_2g_ (Γ) mode respectively^12^. The other peaks at 418 and 343 cm^−1^ are attributed to the out-of-plane A^1^_g_ mode and the in-plane vibrational E^1^_2g_ (M) mode. Studies have previously shown that Raman characterization can provide unambiguous and nondestructive identification of the thickness of WS_2_; the A_1g_ (Γ) mode softens while both the 2LA (M) and E^!^_2g_ (Γ) modes present a subtle red-shift with a decreasing number of layers^5^,^27^. In particular, at 514.5 nm laser excitation, the WS_2_ spectrum reveals a striking increase in the intensity ratio of the 2LA (M) to A_1g_ (Γ) phonon modes due to a double resonance process^27^. Table 1 summarizes the frequency for the three main Raman modes A_1g_ (Γ), 2LA(M) and E^!^_2g_ (Γ), as well as the intensity ratio for the two most intense peaks in our pulsed laser fabricated samples on Ag. Based on these values, it can be concluded that atomically thin WS_2_ films (≤5 layers) can be formed at any laser energy within the range of 50–200 mJ as long as the deposition time was capped at a maximum of 10 seconds. Compared to chemically derived WS_2_^21^,^23^, our samples exhibited Raman peaks with broader full width at half maximums (FWHM), such that it becomes difficult to distinguish the phonon modes in the 260–330 cm^−1^ range without multi-peak Lorentzian fitting. This indicates the presence of defects arising from disorder in the atomic arrangement of the WS_2_ film. In addition, the samples fabricated at a laser energy of 50 mJ demonstrate much lower signal intensity, possibly indicating only partial crystallization or a large concentration of defects. As such, even though the thinnest films are formed at the lowest energy, for an optimal balance between film thickness and crystalline quality, a laser energy of 100 mJ would be most ideal to grow highly crystalline few-layer WS_2_.

Although insulating substrates such as sapphire are preferred substrates for growing 2D materials for characterization of electronic properties, it was found that such substrates do not produce good quality WS_2_ films in pulsed laser synthesis. Figure 1a shows that despite the use of higher laser energies of 200 mJ to fabricate the sapphire sample, its Raman spectrum resembles the partially crystalline and highly defective spectrum of the Ag sample fabricated at 50 mJ and 10 s. At lower energies of 100 and 50 mJ, the characteristic peaks of 2H-WS_2_ are completely absent. This obstacle can be however, overcome by sputtering a very thin layer of the Ag buffer on the substrate. Our investigations reveal that the minimum thickness that the Ag layer can have before it begins to significantly affect the crystalline quality of the overlying WS_2_ film is ~8 nm (Fig. 1c). These WS_2_ films were grown on quartz, which not only permits the direct synthesis of crystalline few-layer WS_2_ on insulating substrates without the need for transfer techniques, but also allows the fabrication of samples that are capable of transmitting visible light. Figure 1d shows that the triple-layered WS_2_ film on quartz has an average optical transmittance of 71% in the wavelength range between 400 and 800 nm. Reducing the buffer layer to such minimal thickness also results in *in-situ* consumption of most, if not all, of the pure Ag metal to form Ag_2_S (a phase which was detected in the TEM, XPS and XRD analysis). This was deduced from the increase in optical transmission in the sample after deposition of WS_2_, also shown in Fig. 1d. The high optical transmittance of our WS_2_ films on quartz means that they are suitable for solar energy applications. The presence of the Ag_2_S phase is also beneficial as the material appears to be a promising solar absorbing material with its narrow band gap of ~0.9 eV^28^ and its unique combination of properties such as light absorbance in the near-infrared spectral regions^29^.

**Figure 2:**
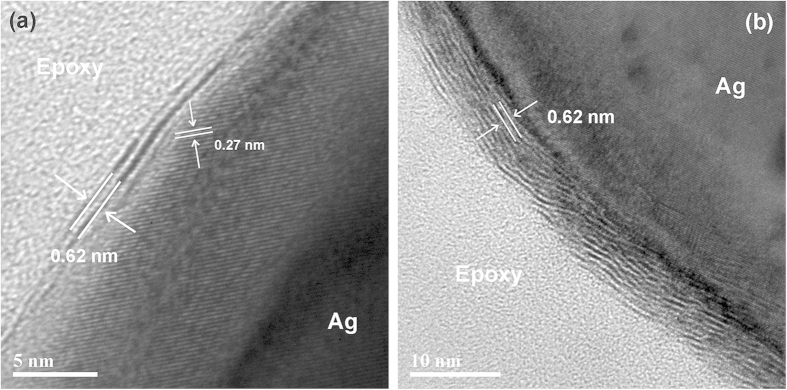
Cross-section TEM of Ag samples fabricated at 50 mJ laser energy and a deposition time of (**a**) 10 seconds and (**b**) 30 seconds.

**Figure 3:**
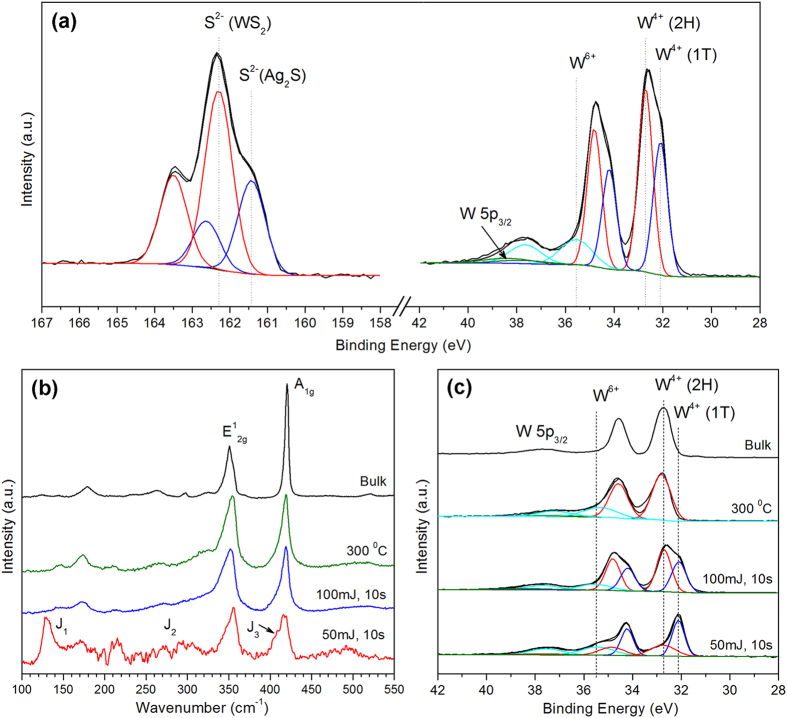
(**a**) XPS spectra showing W 4f and S 2p core level peak regions for as-deposited samples fabricated using 100 mJ laser energy and 10 s deposition time. (**b**) Raman spectra of WS_2_ films deposited on Ag. The J_1_, J_2_, and J_3_ peaks are only active in the as-deposited sample fabricated at 50 mJ and 10 s. The spectrum for WS_2_ films annealed at 300 °C more closely resembles that of bulk 2H-WS_2_. (**c**) XPS spectra showing the W 4f core level peak regions of as-deposited, annealed and bulk samples. W 4f peaks were deconvoluted with 2H (red) and 1T (blue) components. As-deposited WS_2_ sheets at 50 and 100 mJ have 1T-to-2H phase ratio of 1.67 and 0.73 respectively, whereas after annealing at 300 °C the material is converted to purely 2H-WS_2_.

High resolution transmission electron microscope (TEM) images of our pulsed laser fabricated samples are shown in Fig. 2, and reveal the stacking of WS_2_ (002) layers with an interplanar spacing of 0.62 nm on top of silver. Figure 3a provides direct evidence of the successful formation of double-layered WS_2_ in the sample fabricated at 50 mJ and 10 seconds, with the first WS_2_ layer forming covalent bonds to the previously mentioned Ag_2_S phase that develops *in-situ* on the surface of the Ag buffer. This bonding between the two layers manifests as an indistinct boundary at the interface contrary to the sharp interfaces of 2D layered materials grown by van der Waals epitaxy. The formation of the Ag_2_S phase is supported by the presence of lattice fringes with interplanar spacings of 0.27 nm located underneath the WS_2_ film that can be ascribed to the (120) plane of monoclinic acanthite Ag_2_S [JCPDS #14-0072]. This Ag_2_S phase was also previously found to similarly promote the growth of ultrathin MoS_2_ by PLD through lattice matching and conventional epitaxy^25^. Figure 3b shows the change in the WS_2_ film when the deposition time is increased to 30 seconds. The number of layers have increased to >5, verifying the results of the Raman measurements.

To characterize the chemical nature and bonding state of WS_2_ on Ag metal, X-ray photoelectron spectroscopy (XPS) was employed. Figure 3a depicts the W 4f and S 2p core level XPS scans for the WS_2_ film on silver. Two doublets are present in the S 2p spectra, one occurring at 162.3 and 163.5 eV, consistent with the S^2−^ species of WS_2_, while the second pair located at 161.1 and 162.4 eV can be assigned to the S 2p_3/2_ and S 2p_1/2_ peaks of Ag_2_S. The presence of both doublets confirms the successful growth of WS_2_ as well as the *in-situ* formation of Ag_2_S in the samples. For the W 4f spectra, there is a small shoulder at 35.6 eV corresponding to the W^6+^ state that shows the formation of tungsten oxide in the as-deposited films. This oxide can arise in part from surface oxidation of the WS_2_ target, and in part from unreacted W atoms remaining on the substrate surface after completion of the WS_2_ film. These unreacted W atoms are always produced because some S atoms are initially consumed in the making of the Ag_2_S phase. The oxide is believed to form sort of capping layer over the WS_2_ film when it is first exposed to air, and does not negatively affect the quality the film itself as the calculated stoichiometric ratio of S : W is very close to the ideal value of 2. It was also observed that the main tungsten doublet peak can be deconvoluted into two separate pairs. The second doublet (red curve) occurs at binding energies of 32.7 and 34.8 eV, which corresponds well with the W^4+^ species of highly crystalline 2H-WS_2_^30^. The doublet at 32.1 and 34.2 eV (blue curve) on the other hand lies between the binding energies of metallic tungsten (W^0+^) and W^4+^, and appears to be due to a partially sulfided, intermediate W^x+^ state. However, calculation of the stoichiometric ratio of S: W atoms reveal that both W tungsten species (red and blue curves) have a S : W ratio of ~2, indicating that the W 4f doublet located at 32.1 and 34.2 eV is also due to a W^4+^ state. The negative shift of peak binding energies by 0.6 eV is thus believed to be attributed to the formation of the 1T-phase of WS_2_, consistent with its metallic nature, and is comparable to with previous studies on 1T-MX_2_ materials^14^,^31^.

The formation of the 1T-phase can also be established by closer inspection of the Raman spectra, as well as by powder X-ray diffraction (XRD). As noted by Voiry *et al*^14^, the Raman spectra of 1T-WS_2_ display the additional modes of J_1_, J_2_ and J_3_ that are attributed to the superlattice structure of the distorted 1T-phase. However, these Raman modes are only readily observable when the concentration of 1T-WS_2_ dominates over the 2H-phase. Figure 3b shows the Raman results from two of our as-deposited samples with widely differing concentrations of 1T-WS_2_. It is apparent that the J_1_, J_2_ and J_3_ modes are noticeable only in the sample fabricated at 50 mJ and 10 s due to the much higher 1T-to-2H ratio of 1.67. In contrast, the sample fabricated at 100 mJ and 10 s only has a 1T-to-2H ratio of 0.73, and correspondingly the typical Raman peaks of 1T-WS_2_ have almost completely vanished. As-deposited samples were also annealed at varying temperatures to test the stability of the 1T-phase. Included for comparison in Fig. 3b is a sample annealed at 300 °C for 30 minutes, wherein a complete phase transformation from 1T to 2H-WS_2_ was achieved as revealed by XPS (Fig. 3c). The Raman bands of 2H-WS_2_ in this sample appear more distinct; in particular the combination mode of 2LA-E^2^_2g_ at ~320 cm^−1^ becomes visibly noticeable. Nonetheless, the peaks still lack the sharpness of CVD grown WS_2_ sheets, and there is only a slight increase in the peak intensities while the FWHMs remained largely unchanged. This indicates that there is some degree of atomic disorder present even in the pure 2H-phase, and the crystalline quality of PLD grown WS_2_ on Ag is not quite comparable to CVD grown WS_2_ layers. The fact that the 1T to 2H phase transition can be induced with thermal annealing indicates that the 1T-WS_2_ produced in this work is metastable. Indeed, it was observed that partial 1T to 2H transition begins to occur even at temperatures as low as 100 °C, which would be detrimental if the 1T-phase was intended for use at high temperatures. The ability to prepare metastable 1T-WS_2_ layers at temperatures higher than the 1T to 2H transition is rather surprising. However, this is likely to be simply due to the short deposition times and fast cooling rate, such that the 1T-structure does not have sufficient time to relax to the more stable 2H-phase.

**Figure 4:**
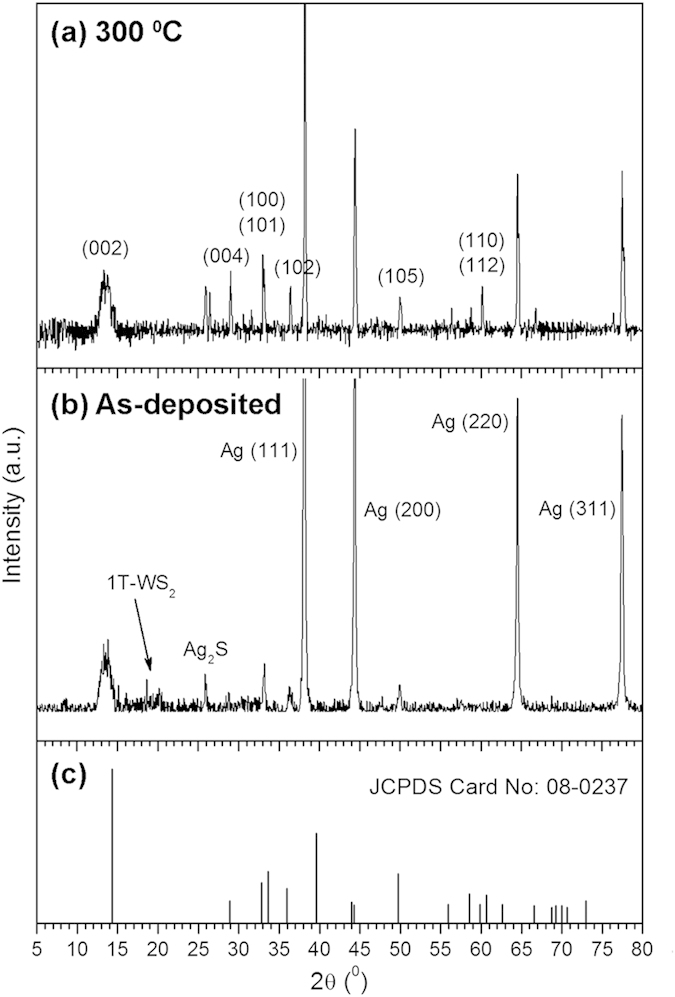
Powder X-ray diffractograms of WS_2_ nanosheets (**a**) annealed at 300 °C and (**b**) as-deposited at 50 mJ and 10 s. (**c**) Bulk diffraction peaks of 2H-WS_2_.

Unlike with 2H-WS_2_, characterization of 1T-WS_2_ by powder XRD is rather challenging because the 1T-phase does not exhibit a well-defined crystalline structure and well-resolved diffraction peaks^31^. This problem is exacerbated by the fact that our samples are all mixtures of 1T and 2H-WS_2_. Nonetheless, we were still able to observe differences in the XRD diffractograms obtained before and after annealing. As shown in Fig. 4, both the as-deposited and annealed samples have peaks corresponding to the known 2H-WS_2_ pattern. In the as-deposited sample, the presence of a broad peak at 14.32° with the highest intensity reveals the preferential growth of WS_2_ sheets along the (002) direction. In contrast, the XRD pattern of the annealed sample shows a more intense (100) peak, which suggests the growth of protrusion edges along the (100) direction. Compared with 2H-WS_2_, the (002) peak of both the as-deposited and annealed sample is shifted to lower 2θ values, indicating a lattice expansion of (002) layers, i.e. 0.62 nm compared to 0.616 nm. The annealed sample also presents additional peaks corresponding to 2H-WS_2_, suggesting an improvement in crystallinity of the WS_2_ film that may be attributed to a 1T to 2H-phase transformation. There is furthermore a very minor peak around 19–20° that vanishes after thermal annealing. As this peak cannot be indexed to either 2H-WS_2,_ Ag, Ag_2_S or even WO_3_, and has previously been observed to be present in 1T-WS_2_^31^, we believe that it can be attributed to the presence of the 1T-phase.

**Figure 5:**
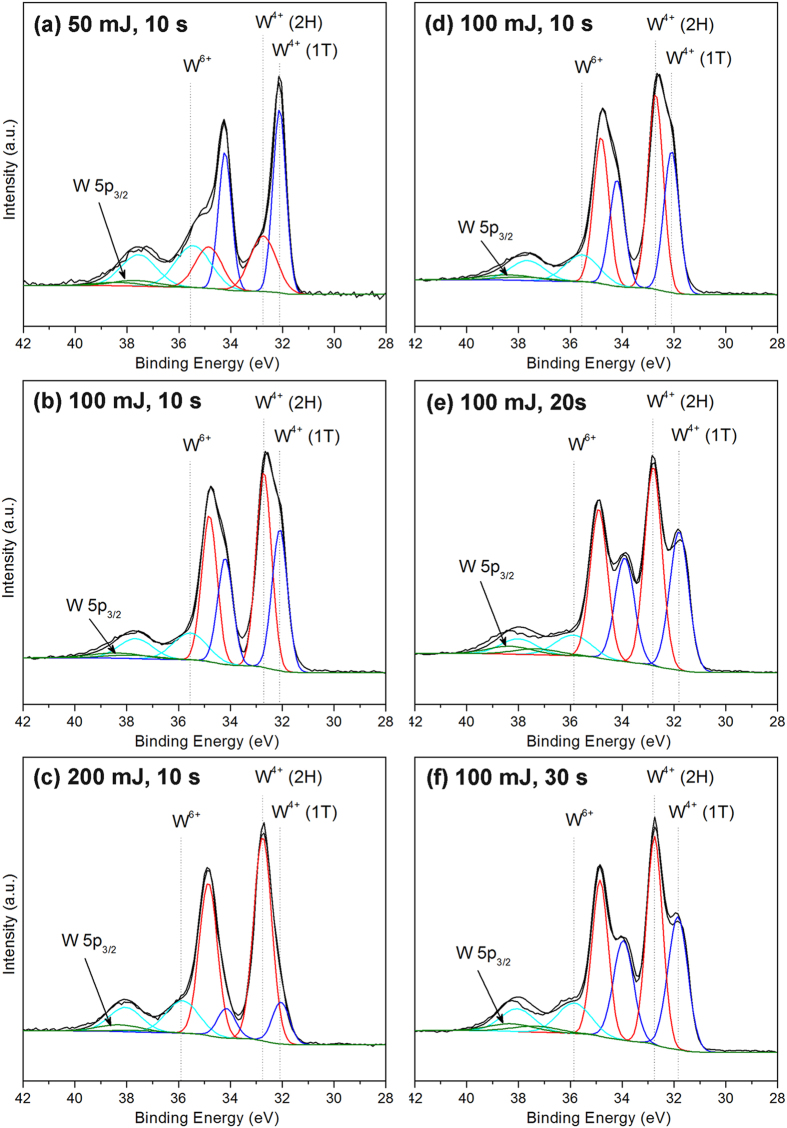
XPS spectra showing W 4f core level peak regions for Ag samples fabricated at different laser energies and deposition times.

It was observed that the concentration of the 1T-WS_2_ phase appears to decrease with longer deposition times and higher laser energies (Fig. 5). In addition, the peak position of the W 4f doublet for 1T-WS_2_ continues to shift to lower binding energies with increasing deposition times, an indication of increasingly metallic character. This phenomenon could possibly be due to bombardment of the growing film by energetic species in the pulsed laser deposition process, which may lead to the breaking of W-S bonds in 1T-WS_2_. It is likely that with longer deposition times, the 1T-WS_2_ phase would convert completely into metallic tungsten. Similarly, the formation of a metallic layer was previously observed during prolonged sputtering of WS_2_^32^, another ion-assisted deposition technique. The breaking of the W-S bond appears to affect only 1T-WS_2_ and not 2H-WS_2_, as seen by the negligible negative shift in the W 4f peak position for the 2H polymorph. This is deduced to be a consequence of the higher thermodynamic stability of 2H-WS_2_, rendering it less likely to be affected by the bombardment of energetic species during film growth.

**Figure 6:**
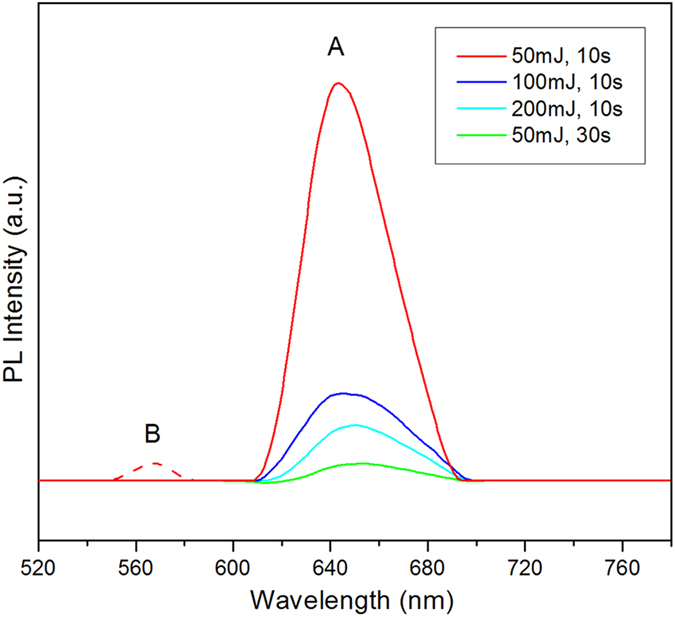
Photoluminescence spectra of as-grown samples fabricated on Ag substrates at λ_ex_ = 488 nm.

The presence of the 1T-phase also affects the photoluminescence (PL) measurements of our samples. Weak PL was observed in as-deposited WS_2_ nanosheets, as expected from their partial metallic character. Enlarging the emission spectra allows us to see that the sample fabricated at 50 mJ and 10 s presents a major peak at ~645 nm and a shoulder peak at ~570 nm (Fig. 6), corresponding to A and B excitonic transitions of the K point of the Brillouin zone. Curiously, the expected intensity and position dependence of the major PL peak with layer thickness can be observed even among the as-deposited samples despite the varying concentrations of 1T-WS_2_. As shown in Fig. 6, the major PL peak at ~640 nm quickly diminishes in intensity and gradually red-shifts with increasing layer thickness, a trend that has been observed by other groups^12^,^22^,^33^. This is unusual as the thinnest sample also has the highest concentration of 1T-WS_2_, which is expected to inhibit PL, and yet this sample gives the highest emission intensity. Because PL originates near the surface of a material, and is sensitive enough to be affected by surface adsorbates, the fact that the PL spectra of as-deposited samples appears to be largely unaffected by the presence of 1T-WS_2_ suggests that the 1T-phase is located away from the surface of the film. While the origin of 1T-WS_2_ in our samples is as yet unclear, we believe that it can be attributed to the capacity of Ag atoms to act as an electron donor for W (owing to Ag having more valence electrons than W). The stabilization mechanism is therefore similar to chemically exfoliated MoS_2_ and WS_2_, wherein the 1T-phase is stabilized by substitutional doping of an electron donating atom. In such a case, the 1T-phase in our samples would be concentrated at the interface while 2H-WS_2_ would dominate at the surface of the film, which could explain their unexpected PL results. Further experimental and theoretical studies would be required to verify these hypotheses.

From the above results, it appears that Ag is indeed capable of stabilizing the 1T-phase of WS_2_, which is noteworthy as recent studies have shown that the 2H and 1T phase have matching lattices and coherent interfaces between domains of the two phases can be formed^34^. As 2H-WS_2_ is a semiconductor and 1T-WS_2_ a metal, localized phase stabilization of the 1T phase to form a 2H-1T hybrid structure presents a viable route to achieving unique electronic heterojunctions across a chemically homogeneous layer, which is advantageous for the fabrication of molecular electronic devices. Furthermore, studies have revealed that the 1T-WS_2_ phase possesses higher reactivity and catalytic ability compared to the 2H-phase^14^,^22^. With Ag also being catalytically active and considered a low-cost alternative to Pt^35^, our findings open up the possibility of Ag stabilized 1T-WS_2_ nanostructures as superior catalysts for the hydrogen evolution reaction. Even so, some challenges remain, not least of which is the impact of the Ag_2_S phase on the intrinsic property of the WS_2_ film. Due to the covalent bonds holding WS_2_ to the buffer layer, it is unlikely that the Ag_2_S phase can be removed without damaging the crystal structure of the WS_2_ sheets. Thus any effort to incorporate pulsed laser deposited WS_2_ into electronic devices must take into account the presence of this Ag_2_S phase, and further fundamental studies on the exact electronic properties of the WS_2_/Ag_2_S material are necessary.

In summary, we have successfully fabricated crystalline few-layer WS_2_ on Ag by pulsed laser deposition. The growth process involves the *in-situ* formation of a lattice matching Ag_2_S phase and that promotes the formation of crystalline WS_2_ through conventional epitaxy rather than expected Van der Waals epitaxy. Samples fabricated at a laser energy of 100 mJ and a deposition time of 10 seconds demonstrated the optimum balance between crystalline quality and film thickness. It was also found that crystalline WS_2_ is able to form on Ag layers as thin as 8 nm, producing samples with high optical transmittance in the visible range, which is advantageous for applications in optoelectronic devices and solar cells. In addition, the Ag substrate was observed to stabilize the 1T-polymorph of WS_2_, resulting in a hybrid 2H-1T structure. Further work would however be required to fully understand the factors behind the stabilization of the 1T phase on Ag before such controlled fabrication of such hybrid or even purely 1T-WS_2_ structures can be achieved.

## Methods

### Synthesis

Ag films of approximately 500 nm thickness were sputtered onto normal doped (n+) Si substrates and loaded in a KrF (λ = 248 nm) Lambda Physik excimer PLD system. During deposition, a WS_2_ target, 99.9% (Inlab Supplies) was ablated at a laser frequency of 10 Hz for a pulse duration of 25 ns in a vacuum environment of 2 × 10^−6^ Torr. The target was rotated at a speed of 6 rpm, with the laser (spot size 1 mm^3^) ablating a circular outline of 2 cm in radius in order obtain a uniform film. The laser energies used for ablation was kept within the range of 50–200 mJ, and the deposition time between 10–30 seconds. Substrate temperature was kept at 450 °C during deposition, and subsequently decreased at a controlled rate of 20 °C/min until the temperature reached 300 °C, thereupon natural cooling processes took over. The fabrication temperature of 450 °C was selected as it is the optimum for growth of ultrathin WS_2_ films by PLD; any lower and the crystalline quality decreases because the atoms do not have sufficient thermal energy to rearrange themselves into a periodic configuration, higher temperatures only provide minimal increase in crystallinity, whereas temperatures  >700 °C promotes the reaction of tungsten with oxygen.

### Characterization

WS_2_ films were characterized by Raman spectroscopy performed in a Horiba MicroRaman HR Evolution System using an Argon laser beam with an excitation wavelength of 514.5 nm.

The phonon mode from silicon at 520.6 cm^−1^ was used for calibration. The Raman spectrum for bulk WS_2_ was collected from the WS_2_ target used. PL measurements were taken in a Perkin-Elmer fluorescence spectrometer LS 55 with excitation wavelength of 488 nm. A JEOL JEM-2010F high resolution transmission electron microscopy (TEM) operated at 200 kV was used to obtain bright field cross-section images of the samples. Surface composition was analyzed by x-ray photoelectron spectroscopy (XPS) using a Kratos Analytical Axis Ultra^DLD^ UHV spectrometer with a monochromatized Al Kα x-ray source (1486.6 eV) scanning a spot size of 700 μm by 300 μm. Core-level XPS spectra were obtained by photoelectrons at a take-off angle of 90°, measured with respect to the sample surface at a vacuum of 5 × 10^−9^  Torr. The pass energy for the narrow scans were set at 10 eV.

## Additional Information

**How to cite this article**: Loh, T. A.J. *et al.* One-step Synthesis of Few-layer WS_2_ by Pulsed Laser Deposition. *Sci. Rep.* **5**, 18116; doi: 10.1038/srep18116 (2015).

## Figures and Tables

**Table 1:** Peak position for the Raman modes A_1g_ (Γ), 2LA(M) and E^!^_2g_ (Γ), as well as the intensity ratio of A_1g_ (Γ) to 2LA(M) for the as-deposited samples on Ag.

**Laser Energy (mJ)**	**Deposition Time**	**A**_**1g**_ **(Γ) (cm**^−**1**^)	**E**^**!**^_**2g**_ **(Γ) (cm**^−**1**^)	**2LA (M) (cm**^−**1**^)	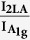	**Estimated Number of Layers**
50	10	418	351	355	0.94	~2
100	10	418.7	350.6	355	0.75	~3
200	10	419.1	350.1	354.9	0.58	4–5
50	30	419.5	350.2	355	0.51	>5
Bulk	–	420	350.4	355.2	0.46	Bulk

## References

[b1] Chhowalla M. *et al.* The chemistry of two-dimensional layered transition metal dichalcogenide nanosheets. Nature Chemistry 5, 263–275 (2013). 10.1038/nchem.1589 23511414

[b2] Wang Q. H., Kalantar-Zadeh K., Kis A., Coleman J. N. & Strano M. S. Electronics and optoelectronics of two-dimensional transition metal dichalcogenides. Nat. Nanotechnol. 7, 699–712 (2012). 23132225 10.1038/nnano.2012.193

[b3] Huang X., Zeng Z. & Zhang H. Metal dichalcogenide nanosheets: preparation, properties and applications. Chem. Soc. Rev. 42, 1934–1946 (2013). 23344899 10.1039/c2cs35387c

[b4] Butler S. Z. *et al.* Progress, challenges, and opportunities in two-dimensional materials beyond graphene. ACS Nano 7, 2898–2926 (2013). 23464873 10.1021/nn400280c

[b5] Gutiérrez H. R. *et al.* Extraordinary room-temperature photoluminescence in triangular WS_2_ monolayers. Nano Lett. 13, 3447–3454 (2013). 23194096 10.1021/nl3026357

[b6] Jo S., Ubrig N., Berger H., Kuzmenko A. B. & Morpurgo A. F. Mono- and bilayer WS_2_ light emitting transistors. Nano Lett. 14, 2019 (2014). 24669957 10.1021/nl500171v

[b7] Bernardi M., Palummo M. & Grossman J. C. Extraordinary sunlight absorption and one nanometer thick photovoltaics using two-dimensional monolayer materials. Nano Lett. 13, 3664 (2013). 23750910 10.1021/nl401544y

[b8] Cheng L. *et al.* Ultrathin WS_2_ nanoflakes as a high-performance electrocatalyst for the hydrogen evolution reaction. Angew. Chem. 126, 7994–7997 (2014). 10.1002/anie.201402315 24838978

[b9] Bhandavat R., David L. & Singh G. Synthesis of surface-functionalized WS_2_ nanosheets and performance as Li-ion battery anodes. J. Phys. Chem. Lett. 3, 1523–1530 (2012). 26285632 10.1021/jz300480w

[b10] Ratha S. & Rout C. S. Supercapacitor electrodes based on layered tungsten disulfide-reduced graphene oxide hybrids synthesized by a facile hydrothermal method. ACS Appl. Mater. Interfaces, 5, 11427 (2013). 24125029 10.1021/am403663f

[b11] Zhu Z. Y., Cheng Y. C. & Schwingenschlögl U. Giant spin-orbit-induced spin splitting in two-dimensional transition-metal dichalcogenide semiconductors. Phys. Rev. B 84, 153402 (2011).

[b12] Zeng H. *et al.* Optical signature of symmetry variations and spin-valley coupling in atomically thin tungsten dichalcogenides. Sci. Rep. 3, 1608 (2013). 23575911 10.1038/srep01608 PMC3622914

[b13] Shi H., Pan H., Zhang Y.-W. & Yakobson B. I. Quasiparticle band structures and optical properties of strained monolayer MoS_2_ and WS_2_. Phys. Rev. B 87, 155304 (2013).

[b14] Voiry D. *et al.* Enhanced catalytic activity in strained chemically exfoliated WS_2_ nanosheets for hydrogen evolution. Nat. Mater. 12, 850–855 (2013). 23832127 10.1038/nmat3700

[b15] Lukowski M. A. *et al.* Highly active hydrogen evolution catalysis from metallic WS_2_ nanosheets. Energy Environ. Sci. 7, 2608 (2014).

[b16] Voiry D. *et al.* Conducting MoS_2_ nanosheets as catalysts for hydrogen evolution reaction. Nano Lett. 13, 6222 (2013). 24251828 10.1021/nl403661s

[b17] Zhao W. J. *et al.* Evolution of electronic structure in atomically thin sheets of WS_2_ and WSe_2_. ACS Nano 7, 791–797 (2012). 23256505 10.1021/nn305275h

[b18] Zeng Z. *et al.* Single-layer semiconducting nanosheets: high-yield preparation and device fabrication. Angew. Chem., Int. Ed. 50, 11093–11097 (2011). 10.1002/anie.201106004 22021163

[b19] Coleman J. N. *et al.* Two-Dimensional Nanosheets Produced by Liquid Exfoliation of Layered Materials. Science 338, 568–571 (2011). 21292974 10.1126/science.1194975

[b20] Matte H. *et al.* MoS_2_ and WS_2_ analogues of graphene. Angew. Chem., Int. Ed. 49, 4059–4062 (2010). 10.1002/anie.201000009 20425874

[b21] Peimyoo N. *et al.* Nonblinking, intense two-dimensional light emitter: monolayer WS_2_ triangles. ACS Nano 7, 10985–10994 (2013). 24266716 10.1021/nn4046002

[b22] Zhang Y. *et al.* Controlled Growth of High-Quality Monolayer WS_2_ Layers on Sapphire and Imaging Its Grain Boundary. ACS Nano 7, 8963–8971 (2013). 24047054 10.1021/nn403454e

[b23] Cong C. *et al.* Synthesis and optical properties of large-area single-crystalline 2D semiconductor WS_2_ monolayer from chemical vapor deposition. Adv. Optical Mater. 2, 131–136 (2014).

[b24] Koh A. T. T., Foong Y. M. & Chua D. H. C. Cooling rate and energy dependence of pulsed laser fabricated graphene on nickel at reduced temperature. Applied Phys. Lett. 97, 114102 (2010).

[b25] Loh T. A. J. & Chua D. H. C. Pulsed laser fabricated few-layer MoS_2_ on silver. Chem. Phys. Lett. 610−611, 284−287 (2014).

[b26] Chen W., Santos E. J. G., Zhu W., Kaxiras E. & Zhang Z. Tuning the electronic and chemical properties of monolayer MoS_2_ adsorbed on transition metal substrates. Nano Lett. 13, 13, 509–514 (2013). 23320793 10.1021/nl303909f

[b27] Berkdemir A. *et al.* Identification of individual and few layers of WS_2_ using raman spectroscopy. Sci. Rep. 3, 1755 (2013).

[b28] Xu Y. & Schoonen M. A. A. The absolute energy positions of conduction and valence bands of selected semiconducting minerals. Am. Mineral. 85, 543−556 (2000).

[b29] Lei Y. *et al.* Hybrid solar cells with outstanding short-circuit currents based on a room temperature soft-chemical strategy: the case of P3HT:Ag_2_S. J. Am. Chem. Soc. 134, 17392−17395 (2012). 23043595 10.1021/ja307521t

[b30] Morrish R., Haak T. & Wolden C. A. Low-temperature synthesis of n-type WS_2_ thin films via H_2_S plasma sulfurization of WO_3_. Chem. Mater. 36, 3986–3992 (2014).

[b31] Mahler B., Hoepfner V., Liao K. & Ozin G. A. Colloidal synthesis of 1T-WS_2_ and 2H-WS_2_ nanosheets: applications for photocatalytic hydrogen evolution. J. Am. Chem. Soc. 136, 14121–14127 (2014). 25220034 10.1021/ja506261t

[b32] Rumaner L. E., Tazawa T. & Ohuchi F. S. Compositional change of (0001) WS_2_ surfaces induced by ion beam bombardment with energies between 100 and 1500 eV. J. Vac. Sci. Technol. 12, 2451 (1994).

[b33] Elias A. L. *et al.* Controlled synthesis and transfer of large-area WS_2_ sheets: from single layer to few layers. ACS Nano 7, 5235–5242 (2013). 23647141 10.1021/nn400971k

[b34] Eda G. *et al.* Coherent atomic and electronic heterostructures of single-layer MoS_2_. ACS Nano 6, 7311–7317 (2012). 22799455 10.1021/nn302422x

[b35] Danilovic N. *et al.* Enhancing the alkaline hydrogen evolution reaction activity through the bifunctionality of Ni(OH)_2_/metal catalysts. Angewandte 51, 12495–12498 (2012). 10.1002/anie.201204842 23129151

